# Diversity and composition of volant and non-volant small mammals in northern Selangor State Park and adjacent forest of Peninsular Malaysia

**DOI:** 10.3897/BDJ.8.e50304

**Published:** 2020-04-07

**Authors:** Kaviarasu Munian, Shahfiz Mohammad Azman, Norhazwani Ahmad Ruzman, Noor Faradiana Md. Fauzi, Alwani Nur Zakaria

**Affiliations:** 1 Zoology Branch, Forest Biodiversity Division, Forest Research Institute Malaysia, Kepong, Selangor, Malaysia Zoology Branch, Forest Biodiversity Division, Forest Research Institute Malaysia Kepong, Selangor Malaysia

**Keywords:** Gading Forest Reserve, Bukit Kutu Forest Reserve, Bukit Tarek Forest Reserve (Extension), Rodentia, Chiroptera, Diversity indices, taxonomic diversity, taxonomic distinctness

## Abstract

Volant and non-volant small mammals from three forest reserves, located inside and outside Selangor State Park, Malaysia, were trapped and documented. A total of five-line transects, each 200 m long and a total of 100 collapsible cage traps, three harp traps and ten mist nets were deployed at each study site to capture rodents and bats species. The presence of 47 species of volant and non-volant mammals was documented with the highest abundant species being *Leopoldamys sabanus* (n = 61). The Family Vespertilionidae was the most diverse, while Muridae was the most abundant species. Diversity indices have shown forest reserves - Gading Forest Reserve (FR) and Bukit Kutu FR – located in the State Park, have a higher species composition than the impaired adjacent forest reserve, Bukit Tarek FR extension. The taxonomic diversity and taxonomic distinctness of the three forest reserves ranged between 2.433 and 2.610, while the taxonomic distinctness values ranged between 2.638 and 2.748. Even though Gading FR recorded the highest number of species diversity, the Chao 1 diversity estimator and the rarefaction accumulation curve indicated that Bukit Kutu comprised more species. Comparisons between other state parks and national parks in Peninsular Malaysia indicated that Selangor State Park indeed harbours relatively more species of small mammals. Northern Selangor State Park and adjacent forest should be recognised as a conservation priority area, although there are comparatively more species harboured in other regions of the State Park. With the current information on fauna diversity, proper management should be formulated to preserve the existing ecosystems in order to ensure the continuity of fauna diversity in Malaysia.

## Introduction

Protected areas are considered to be an essential strategy for habitat and species conservation (Geldmann et al. 2013, Gray et al. 2016). Malaysia has established networks of protected areas to safeguard the global and nationally significant terrestrial biodiversity. In Peninsular Malaysia, at least four major protected area networks, covering approximately 3 million hectares, are managed by various government agencies, such as The Department of Wildlife and National Parks, Johor National Parks Corporation, Perak State Parks Corporation and State Forestry Departments (Ministry of Nature Resource and Environment 2014). Selangor State Park is located in the state of Selangor and is the third-largest national park in Peninsular Malaysia after Taman Negara and Royal Belum State Park (LI 2010). Selangor State Park is a combination of several forest reserves, located adjacent to the backbone of Peninsular Malaysia; the Titiwangsa Range, which runs from Hulu Selangor in the northern tip of Selangor, through Ulu Gombak and into Hulu Langat district. The State Park was gazetted in 2007 under the National Forestry Act 1985 and Enactment 2005 of Selangor. Selangor State Park covers a total area of more than 108,000 ha and is of critical importance to the people of Selangor, Kuala Lumpur and Putrajaya, as it preserves 98% of the water resources for these areas in addition to preserving climatic and ecological balances. It has a wide range of habitats, including lowland, which consists of riverine vegetation, hill dipterocarp forest, lower montane forest and highland montane forest.

The State Park serves as a reservoir for large mammals, including Malayan tapirs, tigers and sun bears, as well as small mammals, such as bats, rodents, pangolins and tree shrews (Lim et al. 1999, Nor et al. 2001, Lim et al. 2009, Sing et al. 2013). Several studies on fauna have been conducted in some parts of the Park (forest reserve), but most of them were concentrated in the central and southern parts, particularly in Ulu Gombak (e.g. Sing et al. 2013) and Hulu Langat districts (e.g. Lim et al. 2009), mainly due to the ease of access to these forest reserves from nearby cities. Nevertheless, to the best of our knowledge, limited studies on small mammals have been performed in the northern region of Selangor State Park, although some of these forest reserves are a relatively large forest complex and an important wildlife reserve. For example, Gading Forest Reserve is the second largest forest reserve in Selangor with a total area of 18,828 ha (Selangor Forestry Department).

The mammal diversity in Malaysia is quite high with at least 440 species of mammals reported (Department of Wildlife and National Parks 2016), of which 15% (66 species) are endemic to Malaysia (Payne et al. 1998, Francis and Barrett 2008). Small mammals, in particular, are the target group of species in this study that describes any mammal that weighs less than 5 kg. The emphasis was on two groups of small mammals, specifically volant and non-volant. The order Chiroptera is the second most diverse of the mammalian orders, exhibiting great physiological and ecological diversity (Hutson et al. 2001). Bats have long been thought to play important ecological roles in prey and predation, arthropod suppression, seed dispersal, pollination, distribution of materials and nutrients and recycling (Kunz et al. 2011). Non-volant small mammals, such as rodents and shrews, are the most common and widespread species that play a significant role in many ecosystems worldwide, including tropical rainforests. These mammals can influence tree recruitment through selective foraging on seeds (García et al. 2005, Zwolak et al. 2010) and seedlings (Ostfeld et al. 1997, Gómez et al. 2003). Rodents and shrews are important consumers of invertebrates (Drożdż 1966, Churchfield and Rychlik 2006) and may control insect populations (Jones et al. 1998). Small mammals are a crucial part of the diet of a number of predatory species and birds of prey (Jędrzejewska and Jędrzejewski 1998).

Basic information on species diversity is essential for park managers and local authorities to develop sound management intent and effect. However, a lack of basic knowledge on biodiversity could lead to non-holistic local planning and would have a negative impact on the environment, especially on fauna diversity. The aims of this study are, therefore, to identify small mammal diversity in the northern region of Selangor State Park and to produce a comprehensive checklist of small mammals in Selangor State Park. Secondly, we intend to compare the composition of small mammals in different i) forest structures both inside and outside the park and ii) national parks and/or state parks located in Peninsular Malaysia.

## Materials and methods

### Study Areas

**Figure 1.**
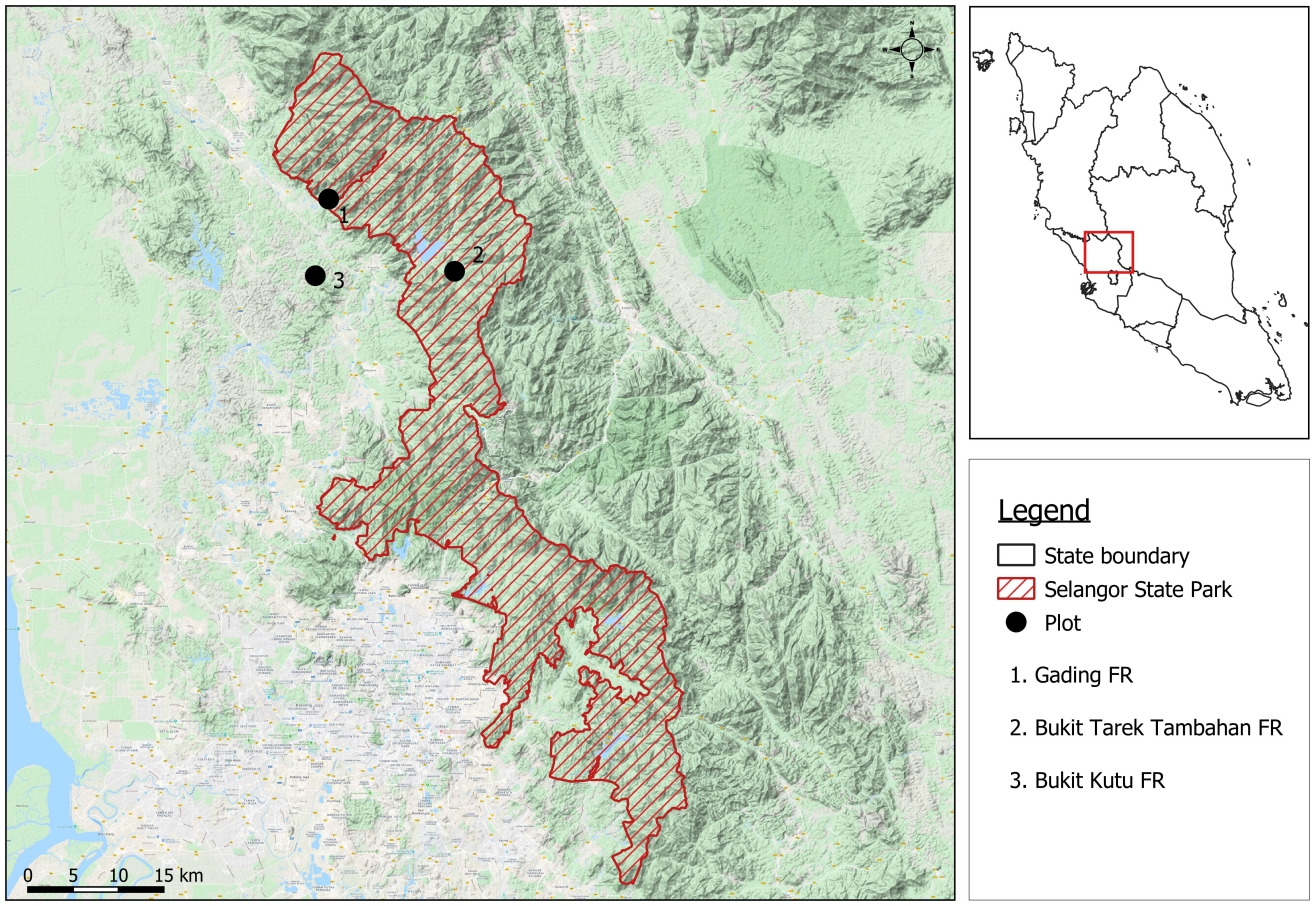
Location of Selangor state Park and present study sites. The black dots represent the location of present study sites.

Two forest reserves, located in the north of Selangor State Park, namely Gading Forest Reserve (FR) and Bukit Kutu FR and one forest reserve outside the park, were selected. Gading FR is one of the largest forests in northern Selangor with a total area of 18,828 ha (Fig. 1). Bukit Kutu FR covered a total area of 11,700 ha and was considered a wildlife sanctuary. Both Gading FR and Bukit Kutu FR are located approximately 20 km apart. Bukit Tarek FR Extension (E) is the third study site. Bukit Tarek FR (E) is an additional forest land to the existing Bukit Tarek FR. It is located south to Gading FR and west to Bukit Kutu FR with a total area of 3,560 ha of forested land. Bukit Tarek FR (E) is located about 10 km from Gading FR and 15 km from Bukit Kutu FR. Bukit Tarek FR (E) is located outside Selangor State Park and is significantly fragmented as the forest is surrounded by agricultural activities, including rubber and oil palm plantations, while Gading FR and Bukit Kutu FR are connected through adjacent forest reserves via Semangkok FR and Hulu Selangor FR. Fig. 1 and Table 1 show the location and land cover of each study site.

### Data collection of small mammal assemblage

A standardised sampling protocol for each site was carried out using the same type, number and arrangement of traps. Sampling sessions were also held in the same area for the same number of days. A total of five-line transects of 200 m, at an altitude of less than 300 m above sea level, were established in each study site. Each line transect was located at least 50 m apart. The sampling sessions were undertaken from 2017 to 2019. Each sampling session comprised of five days of trapping and at least one sampling session was held each month, consisting of a total of seven sampling sessions for each study site.

The trapping activity of small mammals was carried out using several types of traps, including a collapsible trap for non-volant mammals, while harp traps and mist nets were used to capture volant mammals. A total of 100 collapsible cage traps with dimensions of 42 cm × 16 cm × 16 cm were deployed in each line transect and 20 traps were placed along the transect with an interval of 10 m between the two traps. All the traps were baited with oil palm fruit. During the sampling period, the traps were left open all day and checked early in the morning. Any mammal caught was carefully removed and temporarily placed inside a cloth bag before being examined. Meanwhile, three four banks harp traps and 10 mist nets (9 m × 4 m) were placed in suitable and potential fly paths to trap insectivorous and fruit bats, respectively. Harp traps were checked every hour from 7.30 p.m. to 10.30 p.m. while the mist nets were checked every hour from 6.30 a.m. to 11.00 a.m. and then from 7.30 p.m. to 10.30 p.m.

All the small mammals caught were carefully removed from the traps and nets and placed temporarily in a cloth bag. All captured individuals were thoroughly examined and measured as the species identification of volant and non-volant small mammals is largely dependent on specific measurements. The morphologies of each individual, such as body length, hindfoot, ear size, forearm and tail length, were measured to the nearest millimetre and the weight was recorded in grammes. In addition, the sex of each individual (male or female) and reproductive conditions (pregnant or lactating) were noted. Non-volant and volant small mammals were identified using references such as Medway 1983, Khan 1992, Payne et al. 1998, Kingston et al. 2006, Francis and Barrett 2008 and Jayaraj et al. 2012. After all the individuals were carefully examined, most of the species were tagged and released back to their habitat. Only three representative individuals of each species found at each study site were collected and stored in 70% ethanol as voucher specimens. Liver tissues were excised and stored in a vial containing absolute ethanol for further DNA analysis. All specimens were deposited in the Zoological Collection of Forest Research Institute Malaysia (FRIM).

### Data analysis

The species diversity indices, Shannon-Wiener Index (H’), Evenness (SI) and Dominance (D) were calculated using the Paleontological Statistics (PAST) software (Hammer et al. 2001). The Shannon Diversity Index was used as a relatively reliable method of measuring species diversity (Gotelli and Colwell 2001, Colwell 2009). Species accumulation curves were calculated using iNEXT Online (Hsieh et al. 2016) to infer the completeness of the inventory for small mammal assemblages in the three study sites. The non-parametric t-test was assessed for differences in the abundance of volant and non-volant small mammals using the PAST programme. The Bray-Curtis Similarity Index was calculated to show the similarity in the composition of small mammals between forest structures inside and outside of the park.

We selected classic taxonomic measures developed by Clarke and Warwick (1999), taxonomic diversity and taxonomic distinctness, which measured merged information relative to taxonomy, number of species and evenness of the sample and these remained insensitive to the sampling efforts (Clarke and Warwick 1999). Given the multivariate nature of the information included in this index (Somerfield et al. 1997), it is expected to be more sensitive to anthropogenic disturbances (Warwick and Clarke 1998, Leonard et al. 2006, Marcantonio et al. 2013), such as forest fragmentation.

All the published data on small mammals conducted along Selangor State Park and other national/state parks were gathered to produce a complete checklist. All data were carefully examined and species nomenclature was updated with current naming. The Sorensen-Dice Analysis was calculated to show how similar Selangor State Park is with other national/state parks in Peninsular Malaysia, based on presence data of small mammals.

## Results

In all, 200 and 233 individuals of non-volant and volant small mammals, respectively, were caught in 11,865 trap-nights during the survey. Overall trap success was one animal captured on average for each 27 trap-nights or > 6%. Out of 11,865 trap nights, 199, 142 and 92 (total individuals = 433) small mammals were trapped in Gading FR, Bukit Kutu FR and Bukit Tarek FR (E), respectively (Table 2). These included 31 volant and 16 non-volant small mammal species. Five families of volant small mammals, including Hipposideridae, Nycteridae, Pteropodidae, Rhinolophidae and Vespertilionidae, were documented, while non-volant species originated from the families of Erinaceidae, Muridae, Sciuridae, Soricidae and Tupaiidae. Amongst these families, Vespertilionidae was the most diverse with 12 species, followed by Muridae with nine species. Only one species has been recorded under the Erinaceidae, Tupaiidae and Nycteridae families.

A compilation of small mammal species in Selangor State Park, based on previous studies (e.g. Lim et al. 1999, Lim et al. 2009, Nor et al. 2001, William-Dee et al. 2019,Sing et al. 2013) and the present study, was produced (Table 3). A total of 113 volant and non-volant small mammals have been recorded from these three regions. A comparison was made of small mammals assemblages of Selangor State Park with four other national/state parks found in Peninsular Malaysia, namely, Taman Negara National Park (Kuala Atok and Kuala Tahan) (Siti-Hawa et al. 1985, Lim et al. 1989, Tingga et al. 2012), Royal Belum State Park (Tamrin et al. 2008, Shahfiz et al. 2019), Endau-Rompin State Park (Aihara et al. 2016) and Hulu Terengganu ([Bibr B5570085][Bibr B5570074]). The highest number of small mammal assemblages recorded was in Selangor State Park with a total number of 113 species, followed by Endau-Rompin State Park with 105 species and Hulu Terengganu FR with a record of 74 species (Suppl. material 1).

The long-tailed giant rat (*Leopoldamys sabanus)* was the most abundant rodent species found with a total of 61 (14.1%) individuals, while the least abundant individuals were *Echinosorex gymnura*, *Sundasciurus lowii*, *Rhinosciurus laticaudatus*, *Hipposideros armiger*, *Rhinolophus acuminatus*, *Rhinolophus robinsoni*, *Rhinolophus sedulous*, *Kerivoula minuta*, *Myotis muricola*, *Myotis ridleyi*, *Kerivoula pellucida*, *Kerivoula intermedia*, *Murina cyclotis* and*Tylonycteris pachypus*. These 15 species of volant and non-volant mammals were very scarce, as only one individual each was captured for the entire study period (Table 2). The results also showed that Gading FR was the richest in species (33 species) compared to Bukit Kutu FR (28 species) and Bukit Tarek FR (E) (24 species) (Table 4). The Mann-Whitney U-test showed no significant differences in abundance amongst the three study sites for non-volant small mammals; however, the volant small mammal abundance in Bukit Tarek FR (E) was significantly lower than in the two other areas (Bukit Tarek FR (E) × Gading FR: *u* = 313, p = 0.013; Bukit Tarek FR (E) × Bukit Kutu FR: *u* = 309.5, p = 0.01). Inclusively, the abundance of small mammal assemblages in Bukit Tarek FR (E) is significantly lower than Gading FR (*u* = 840, p = 0.038), but there was no significant difference in the abundance of small mammal assemblages recorded in Bukit Kutu FR and Gading FR.

**Figure 2.**
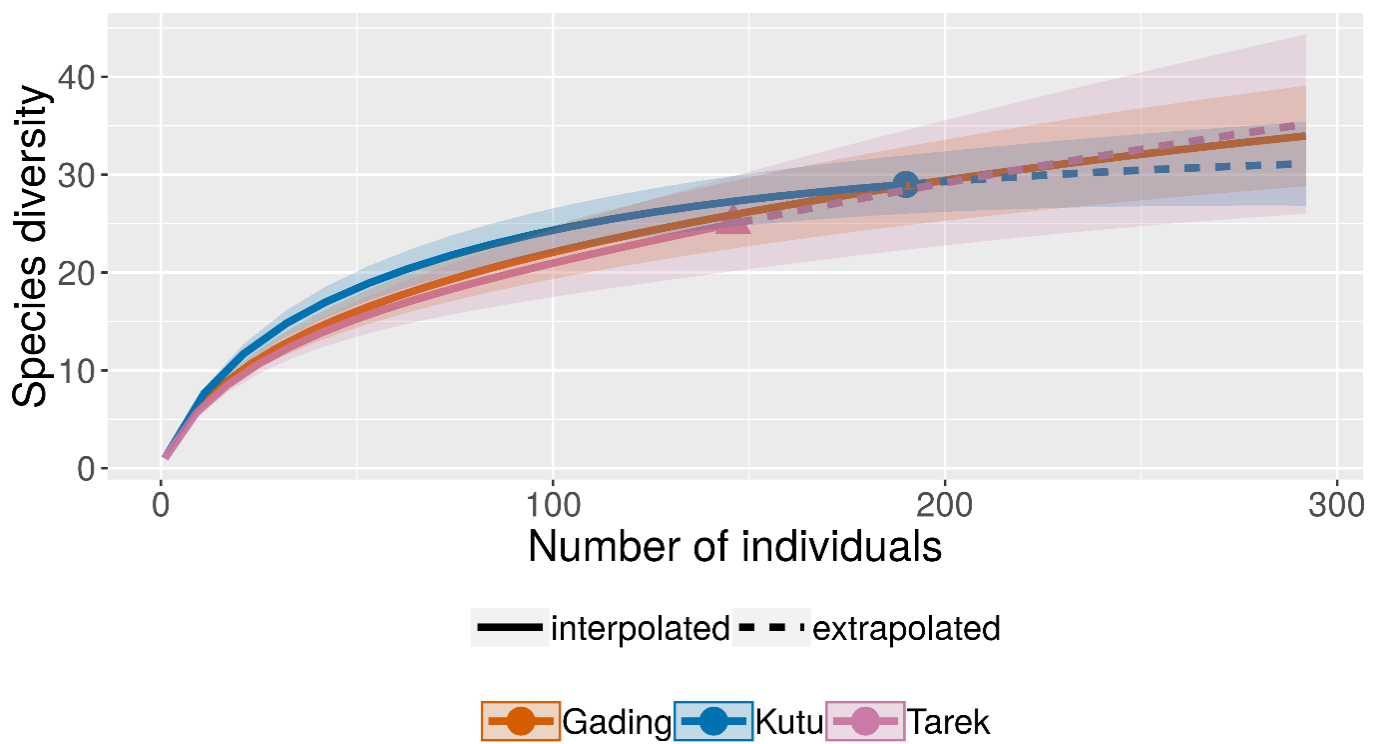
Small mammal accumulation curve, based on rarefaction for each study site in Northern Selangor State Park. Orange line: Gading FR; blue line: Bukit Kutu FR; pink line: Bukit Tarek (E) FR.

A comparison of small mammal species richness accumulation curves for the three study sites (Fig. 2) showed that Gading FR was the richest in rarefaction-estimated species; however, the Chao 1 estimator indicated that Bukit Tarek FR (E) was the richest area (Table 4). Interestingly, Bukit Kutu FR exhibited the highest diversity index values compared to Gading FR and Bukit Tarek FR (E), although Gading FR had more species richness. The results of taxonomic diversity and distinctness showed Bukit Tarek FR (E) with the lowest qualitative data values (Δ*; Table 5).

**Figure 3.**
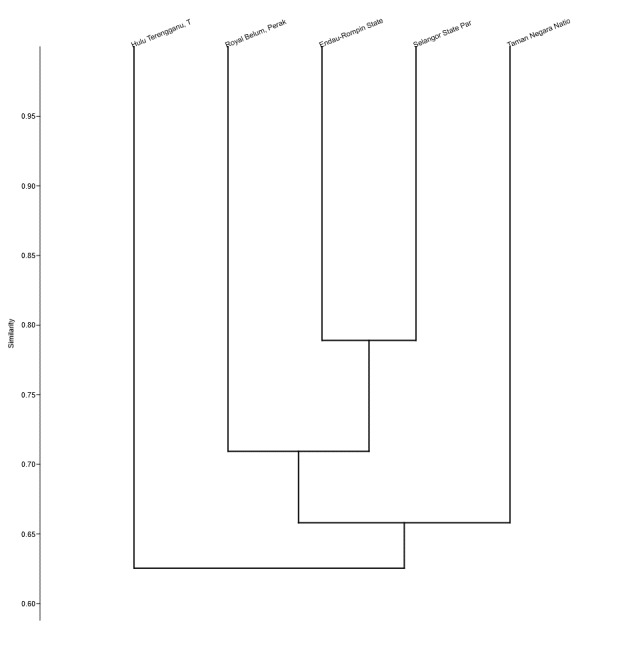
Dendrogram of species similarity between Selangor and other national and/or state parks in Peninsular Malaysia.

The Bray-Curtis and Sorensen similarity coefficients indices ranged from 0 to 1 (0 means no similarity, while 1 means total similarity). Bukit Tarek (E) FR and Bukit Kutu FR had the highest similarity value at 0.512 while Gading FR and Bukit Tarek (E) FR had the lowest value at 0.391 (not shown). The similarity dendrogram of small mammals assemblages in Selangor State Park and four national/state parks in Peninsular Malaysia indicated that Selangor State Park is grouped with Endau-Rompin State Park and Royal Belum State Park at values ranging from 0.744-0.788, whilst the similarity of the composition of Taman Negara National Park and Hulu Terengganu showed values of 0.635 and 0.641, respectively (Fig. 3).

## Discussion

### Diversity of small mammal assemblage

Through this study, we have managed to document a total of 34 species of volant small mammals mainly consisting of bats and 16 species of non-volant small mammals which comprised of rodents. Of this, 24 species of insectivore bats were recorded in this study and these species were mainly recorded in Gading FR (21 species) and Bukit Kutu FR (20 species). This can mainly be the result of the behaviour of insectivorous bats that forage understorey in lowland forest and some of these bats are known to forage in groups (Payne et al. 1998; Tingga et al. 2012). Our results suggested that the family Vespertilionidae is the most diverse, while the family Muridae is the most abundant in three study areas. Only two species of Vespertilionidae were caught in Bukit Tarek FR (E), namely, *Kerivoula papillosa* and *Murina suilla* (Table 2). The main factor that contributes to fewer Vespertilionidae species in Bukit Tarek (E) FR lies within the habitat itself. Bukit Tarek (E) FR is considered a fragmented forest and is surrounded by rubber and oil palm plantations (refer to Table 1). Palaeotropical bat assemblages are dominated by members of the families Rhinolophidae and Hipposideridae and the Vespertilionidae subfamilies, Kerivoulinae and Murininae and many of them are typically highly adapted for foraging in the clutter of the forest interior (‘narrow-space’) (Schnitzler and Kalko 2001; Tingga et al. 2012). Consequently, these species may be more sensitive to forest loss and exhibit greater avoidance of disturbed and open habitats (Kingston et al. 2003). Due to this dependence on forest, these species were probably adversely affected by deforestation and other forest disturbance events (Lane et al. 2006; Struebig et al. 2008). A significant low abundance of volant small mammals in Bukit Tarek (E) FR deceptively exhibits the diversity of these mammals as other contributing factors, such as limited food resources and high predation, might influence the presence of bats generally (Hodgkison et al. 2004, Popa-Lisseanu and Voigt 2009).

On the other hand, the family Muridae has shown that it is relatively abundant in all three study sites, despite the lack of significant differences in abundance amongst the study areas. Small mammals, particularly non-volant small mammals, have a distinct habitat specialisation and can be classified as forest and open land specialists and habitat generalists, each responding differently to changes in landscape complexity (Gentili et al. 2014). However, a relatively similar diversity of non-volant small mammals was achieved across all study areas despite some changes in the microhabitat structure. A similar finding has also been reported by Nakagawa et al. (2006), where the results showed no variations in the abundance of small mammals present in five different habitat types comprising of primary forest and degraded areas in Sarawak, Malaysia.

Species characteristic may be one of the reasons that derive a similar structure of non-volant small mammals from these study areas. The long-tailed giant rat (*Leopoldamys sabanus*) was the most abundant species captured in all three study sites and is present throughout the Sunda region of Southeast Asia. It is a common, generalistic species in local assemblages of small mammals (Lim 1970). The abundance of *L. sabanus* is higher in Bukit Tarek FR (E) relative to the least-impaired habitat in Bukit Kutu FR. Analysis of habitat utilisation by *L. sabanus*, based on spool-and-line-tracks of Wells et al. (2008), revealed *L. sabanus* has a strong ability to move between various forest matrices, such as logged and unlogged forests. A higher abundance of *L. sabanus* in Bukit Tarek (E) proves that the species can forage for its resources in different habitats, including oil palm and rubber plantations and may encourage a healthy reproduction.

Fig. 2 shows the rarefaction curves of species diversity in the three study areas. Although Gading FR had more captures (199 individuals) than all the sites surveyed in this study, Bukit Kutu FR showed the highest species diversity as it had the steepest curve between the other sites. The total sampling effort was still inadequate to document the total diversity of small mammals in Gading FR and Bukit Tarek FR (E) as the cumulative curve was still exponential, but as for Bukit Kutu FR, the curve is approaching the asymptote, indicating the species diversity in the forest reserve may have been successfully identified. The highest species richness recorded in Gading FR is apparent, as Gading FR encompasses a significantly larger area than Bukit Kutu FR and Bukit Tarek FR (E). Areas increase diversity as a larger plot is likely to have more habitats and functions in order to support a greater diversity of species. Besides, many species require a wide range for adequate prey or seed forage. Conversely, the diversity indices have shown that Bukit Kutu FR has the relatively highest diversity values similar to the accumulation curve in Fig. 2. Bukit Kutu FR has the highest Shannon and Evenness values as rarer species have been detected and the abundance of species has been evenly distributed compared to the other two study sites. Although Gading FR was considered to be the richest in the total number of species, there was a decreased discrepancy between the species richness values estimated by the rarefaction and the Chao 1 estimators. These results indicate that Bukit Kutu FR is potentially the richest study site, followed by Gading FR. According to Rabosky (2009), different ecological limits for clades seem to be the main determinants of diversification and, therefore, species richness. Although Bukit Tarek FR (E) recorded the lowest values for both abundance and diversity compared to the other two sites, the differences between all three study areas were found not to be significant (not shown). Such a result in Bukit Tarek FR (E) may be affected by the habitat itself, where the relative abundance, referred to in Pardini et al. (2005) and Cabrini et al. (2013), may be more negatively sensitive to forest fragmentation and isolation than to species richness.

The low species taxonomic distinctness (Δ*) for Bukit Tarek FR (E) also highlights possible forest fragmentation effects in the area; the taxonomic distinctness is quite sensitive for the discriminating species of the affected areas (Salas et al. 2006, da Silva and Batalha 2006). Ricklefs (2001) provided evidence that diversity differences between clades reflect the region’s capacity to support species of a diverse balance of clade diversity. Furthermore, Clarke and Warwick (2001) suggested an equability influence on the Δ index. Bukit Tarek FR (E) was estimated to have an increased number of dominant taxa compared to the other two study areas. Based on the species identified in Bukit Tarek FR (E), the species diversity is justified by the predominance of two major families of Muridae and Pteropdidae, the abundance of which is mainly contributed only by several species (see Table 2).

Based on the similarity dendrogram, it is possible to hypothesise that species composition in Selangor State Park is most similar to those in Endau-Rompin State Park which is followed by Royal Belum State Park. Endau-Rompin is known to support a high diversity of mammals as reported by Aihara et al. 2016 and the small mammals caught from Selangor State Park have a high similarity of almost 80% to the composition of small mammals in Endau-Rompin State Park. The similarity in species richness amongst Selangor State Park, Endau-Rompin State Park and Royal Belum State Park can also be explained due to the proximity of these sites to each other in the west coast of Peninsular Malaysia.

### Conservation priority area

Comparisons were made with the current study representing the northern region of Selangor State Park with Ulu Gombak FR (Sing et al. 2013, William-Dee et al. 2019) as the central region and Hulu Langat FR (refer Lim et al. 2009) at the southern part of the Park (Table 2). Unlike the central and southern regions of the Park, this study is the first attempt to document the small mammal diversity, while the latter studies have been well-studied for several decades. By comparison, both the central and southern regions of the Park have more species than the northern regions; however, the present study shows that there is a wide variety of small mammals inhabiting these habitats. Previous studies have provided comprehensive documentation of mammal diversity; the current study lacks the inclusion of other classes of mammal species, such as medium-sized mammals and large mammals.

The present study is far from reflecting the actual diversity found in the areas studied. Conservation priority should be given, however, as these forest reserves are important habitats for fauna as a whole. In addition, areas that are impaired and fragmented, such as Bukit Tarek FR (E), should also be treated as equally important habitat for fauna. Although Bukit Tarek FR (E) is considerably fragmented and surrounded by plantations, the habitat still provides refuges for moderate volant and non-volant small mammal diversity, which indicates that habitat patches are relevant to include as part of the conservation priority area.

## Conclusions

Our study has shown that there is a total of 47 small mammal species recorded in northern Selangor State Park. Overall, Selangor State Park has a high diversity of small mammals with approximately 96 species. Although the non-protected area and patchy habitat differed greatly from the protected area in terms of species richness, it still appears to have an important role to play in providing habitat for highly-adaptable species. Therefore, it is crucial for the authorities to properly manage these non-protected areas as they continue to function as an ecosystem.

## Supplementary Material

94F8FD38-9538-5249-8FDA-EEAF21CE6BD9 10.3897/BDJ.8.e50304.suppl1 Supplementary material 1 Comparison of small mammals diversity in Selangor State Park, Taman Negara National Park, Royal Belum State Park, Endau-Rompin State Park and Hulu Terengganu Data type checklist of small mammals assemblages in Selangor State Park File: oo_387488.xlsx https://binary.pensoft.net/file/387488 Kaviarasu Munian

## Figures and Tables

**Table 1.** Location and land cover composition of three study sites, based on information fromdata from the European Space Agency 2015 and reclassified, based on IPCC categories.

**Study Site**	**Coordinates**		**Land Cover Composition (%)**		
	**Lat**	**Long**	**Forest**	**Water body**	**Agricul**
Gading FR	101°36’49.85”E	03°37’48.04”N	98.6	0.7	0.68
Bukit Tarek FR (E)	101°36’02.42”E	03°33’13.70”N	55.82	0.0	44.18
Bukit Kutu FR	101°44’19. 52”E	03°33’30.10”N	99.5	0.0	0.5

**Table 2.** List of species and abundance of small mammal assemblage in three study sites.

**No**	**Group**	**Family**	**Species**	**Study Site**			
				**Bukit Tarek (E)**	**HS Gading**	**Bukit Kutu**
1	Non-volant	Erinaceidae	*Echinosorex gymnura*			1
2		Muridae	*Maxomys rajah*	7	8	5
3			*Niviventer cremoriventer*	1	2	
4			*Maxomys whiteheadi*	3	23	13
5			*Leopoldamys sabanus*	17	34	10
6			*Sundamys muelleri*	6	5	4
7			*Rattus tiomanicus*	3		
8			*Maxomys surifer*	5		12
9			*Rattus rattus*	2	2	
10			*Rattus exulans*	1	1	
11		Sciuridae	*Callosciurus notatus*	7	11	
12			*Sundasciurus lowii*	1		
13			*Rhinosciurus laticaudatus*		1	
14		Soricidae	*Crocidura monticola*		2	
15			*Crocidura malayana*		1	1
16		Tupaiidae	*Tupaia glis*	1	4	2
17	Volant	Hipposideridae	*Hipposideros cervinus*	1	23	2
18			*Hipposideros diadema*	1	3	5
19			*Hipposideros larvatus*		7	17
20			*Hipposideros bicolor*	1	6	3
21			*Hipposideros cineraceus*			2
22			*Hipposideros armiger*	1		
23		Nycteridae	*Nycteris tragata*	1		
24		Pteropodidae	*Cynopterus brachyotis*	7	1	14
25			*Balionycteris maculata*	15	1	13
26			*Chironax melanocephalus*		8	2
27			*Rousettus amplexicaudatus*		2	
28			*Cynopterus horsfieldii*			2
29			*Penthetor lucasi*	1	2	3
30		Rhinolophidae	*Rhinolophus trifoliatus*	6	12	4
31			*Rhinolophus affinis*	1	25	10
32			*Rhinolophus acuminatus*		1	
33			*Rhinolophus luctus*		4	
34			*Rhinolophus robinsoni*		1	
35			*Rhinolophus sedulus*			1
36		Vespertilionidae	*Kerivoula papillosa*	2	2	3
37			*Murina suilla*	1		3
38			*Kerivoula minuta*		1	
39			*Kerivoula hardwickii*		1	4
40			*Myotis muricola*		1	
41			*Pipistrellus javanicus*		2	
42			*Myotis ridleyi*		1	
43			*Kerivoula pellucida*		1	
44			*Kerivoula intermedia*			1
45			*Murina cyclotis*			1
46			*Tylonycteris pachypus*			1
47			*Tylonycteris robustula*			3
**Total of Indviduals**				92	199	142

**Table 3.** A compilation of small mammals in Selangor State Park, based on three regions: a:present study and Lim et al. 1999; b:Nor et al. 2001and Sing et al. 2013; c:Lim et al. 2009.

**No**	**Group**	**Family**	**Species**	**Selangor State Park**		
				**Northern^a^**	**Middle^b^**	**Southern^c^**
1	Non-volant	Erinaceidae	*Echinosorex gymnura*	/		/
2			*Hylomys suillus*	/		
3		Muridae	*Bandicota indica*		/	
4			*Berylmys bowersi*	/		
5			*Chlropodamys gliroides*	/	/	
6			*Lenothris canus*	/		
7			*Leopoldamys sabanus*	/	/	/
8			*Maxomys rajah*	/	/	/
9			*Maxomys surifer*	/	/	/
10			*Maxomys whiteheadi*	/	/	/
11			*Niviventer cremoriventer*	/	/	/
12			*Pithecheir parvus*	/		
13			*Rattus exulans*		/	
14			*Rattus rattus*	/	/	
15			*Rattus tiomanicus*		/	/
16			*Sundamys muelleri*	/	/	/
17		Sciuridae	*Callosciurus caniceps*	/	/	/
18			*Callosciurus nigrovittatus*	/		/
19			*Callosciurus notatus*	/	/	/
20			*Callosciurus presvostii*	/		
21			*Lariscus insignis*	/	/	/
22			*Ratufa affinis*	/		
23			*Ratufa bicolor*	/		/
24			*Rhinosciurus laticaudatus*	/		/
25			*Sundasciurus hippurus*	/		
26			*Sundasciurus lowii*	/		/
27			*Sundasciurus tenuis*	/		/
28		Soricidae	*Chimarogale himalayica*	/		
29			*Crocidura fuliginosa*	/		
30			*Crocidura malayana*	/		
31			*Crocidura monticola*	/	/	
32			*Suncus etruscus*	/	/	
33		Tupaiidae	*Ptilocercus lowii*	/		/
34			*Tupaia glis*	/		
35			*Tupaia minor*	/		/
36		Pteromyidae	*Aeromys tehphromelas*	/		
37			*Hylopetes spadiceus*	/		
38			*Iomys horsefieldii*	/		
39			*Petaurista petaurista*	/		
40			*Petinomys setosus*	/		
41			*Pteromyscus pulverulentus*	/		
42	Volant	Hipposideridae	*Hipposideros cervinus*	/	/	/
43			*Hipposideros diadema*	/	/	/
44			*Hipposideros larvatus*	/	/	/
45			*Hipposideros bicolor*	/	/	/
46			*Hipposideros cineraceus*	/	/	
47			*Hipposideros armiger*			/
48			*Coelops frithii*	/	/	
49			*Hipposideros galeritus*	/	/	/
50			*Hipposideros sabanus*		/	
51			*Hipposideros atrox*		/	
52			*Hipposideros redleyii*		/	/
53		Nycteridae	*Nycteris tragata*		/	
54			*Nycteris javanica*	/	/	/
55		Pteropodidae	*Cynopterus brachyotis*	/	/	/
56			*Balionycteris maculata*	/	/	/
57			*Chironax melanocephalus*	/	/	/
58			*Rousettus amplexicaudatus*	/	/	
59			*Cynopterus horsfieldii*	/	/	/
60			*Dyacopterus spadiceus*	/	/	
61			*Eonycteris spelaea*	/	/	/
62			*Macroglossus minimus*	/	/	
63			*Macroglossus sobrinus*	/	/	/
64			*Megaerops ecaudatus*	/	/	/
65			*Macroglossus lagochilus*	/	/	
66			*Pteropus vampyrus*	/	/	
67			*Penthetor lucasi*	/	/	/
68		Rhinolophidae	*Rhinolophus trifoliatus*	/	/	
69			*Rhinolophus affinis*	/	/	/
70			*Rhinolophus acuminatus*	/		
71			*Rhinolophus luctus*	/	/	/
72			*Rhinolophus robinsoni*	/		
73			*Rhinolophus sedulus*	/	/	
74			*Rhinolophus refulgens*		/	
75			*Rhinolophus stheno*	/	/	/
76			*Rhinolophus coelophylus*	/	/	
77			*Rhinolophus lepidus*	/	/	/
78			*Rhinolophus macrotis*			/
79		Vespertilionidae	*Kerivoula papillosa*	/	/	/
80			*Murina suilla*	/	/	/
81			*Murina aenea*		/	
82			*Murina peninsularis*		/	
83			*Kerivoula minuta*	/	/	/
84			*Kerivoula hardwickii*	/	/	
85			*Myotis muricola*	/	/	/
86			*Pipistrellus javanicus*	/		
87			*Pipistrellus stenopterus*		/	
88			*Pipistrellus tenuis*	/		/
89			*Myotis ridleyi*	/	/	
90			*Myotis montivagus*		/	
91			*Myotis horsefieldii*		/	
92			*Kerivoula pellucida*	/	/	
93			*Kerivoula intermedia*	/	/	/
94			*Murina cyclotis*	/	/	
95			*Tylonycteris pachypus*	/	/	
96			*Tylonycteris robustula*	/	/	/
97			*Miniopterus schreibersii*		/	
98			*Eptesicus circumdatus*		/	
99			*Glischropus tylopus*	/	/	/
100			*Hesperoptenus doriae*		/	
101			*Hessperoptenus tomesi*		/	
102			*Hesperoptenus blanfordi*		/	
103			*Philetor brachypterus*		/	
104			*Phoniscus atrox*		/	
105			*Scotophilus kuhlii*		/	
106		Emballonuridae	*Emballonura monticola*	/	/	
107			*Taphozous melanopogon*	/	/	
108			*Taphozous saccolainus*		/	
109		Megadermatidae	*Megaderma lyra*	/	/	
110			*Megaderma spasma*	/	/	/
111		Molossidae	*Cheiromeles torquatus*	/	/	
112			*Tadarida mops*			
113			*Chaerephon* sp.		/	

**Table 4.** Species abundance, richness and diversity values estimated for each study locality.

**Sites**	**Species**	**Individuals**	**Dominance(D)**	**Shannon(H)**	**Evenness**	**Chao 1**
Bukit Tarek (E) FR	24	92	0.09405	2.69	0.6136	46
Gading FR	33	199	0.08644	2.828	0.5125	41.25
Bukit Kutu FR	28	142	0.06626	2.957	0.6873	30.5

**Table 5.** Descriptive statistics of Taxonomic Diversity and Taxonomic Distinctness in three study sites.

**Study Sites**	**Taxonomic diversity Δ**			Taxonomic distinctness Δ*		
	**calculated**	**lower limit**	**upper limit**	**calculated**	**lower limit**	**upper limit**
Gading FR	2.610	2.572	2.643	2.803	2.743	2.789
Bukit Tarek (T) FR	2.433	2.522	2.682	2.638	2.708	2.818
Bukit Kutu FR	2.580	2.565	2.650	2.748	2.734	2.795
